# Occurrence and Distribution of Antibiotic-resistant Bacteria and Transfer of Resistance Genes in Lake Taihu

**DOI:** 10.1264/jsme2.ME13098

**Published:** 2013-11-16

**Authors:** Qian Yin, Dongmei Yue, Yuke Peng, Ying Liu, Lin Xiao

**Affiliations:** 1 State Key Laboratory of Pollution Control and Resource Reuse, School of the Environment, Nanjing University, Nanjing 210023, P.R. China

**Keywords:** antibiotic resistance, horizontal gene transfer, integrons, β-lactamase gene, Lake Taihu

## Abstract

The overuse of antibiotics has accelerated antibiotic resistance in the natural environment, especially fresh water, generating a potential risk for public health around the world. In this study, antibiotic resistance in Lake Taihu was investigated and this was the first thorough data obtained through culture-dependent methods. High percentages of resistance to streptomycin and ampicillin among bacterial isolates were detected, followed by tetracycline and chloramphenicol. Especially high levels of ampicillin resistance in the western and northern regions were illustrated. Bacterial identification of the isolates selected for further study indicated the prevalence of some opportunistic pathogens and 62.0% of the 78 isolates exhibited multiple antibiotic resistance. The presence of ESBLs genes was in the following sequence: *bla_TEM_* > *bla_SHV_* > *bla_CTMX_* and 38.5% of the isolates had a class I integrase gene. Of all tested strains, 80.8% were able to transfer antibiotic resistance through conjugation. We also concluded that some new families of human-associated ESBLs and AmpC genes can be found in natural environmental isolates. The prevalence of antibiotic resistance and the dissemination of transferable antibiotic resistance in bacterial isolates (especially in opportunistic pathogens) was alarming and clearly indicated the urgency of realizing the health risks of antibiotic resistance to human and animal populations who are dependent on Lake Taihu for water consumption.

During the past few decades, antibiotics have been widely used in human clinics, animal husbandry and aquaculture, aiming to fight bacterial infections. The unmonitored and continued use of antibiotics has led to significant antibiotic contamination of diverse environments, generates an increasing selective pressure on microorganisms and consequently increases the prevalence of antibiotic resistance (AR) among bacteria (41). AR has been recognized as a worldwide ecological problem (33) and a significant concern to public health (58). Antibiotic-resistant bacteria (ARB) and associated antibiotic resistance genes (ARGs) are gradually becoming considered as environmental contaminants (3). ARB and ARGs no longer strictly occur in so-called point sources with antibiotic contamination, *e.g.* hospitals, sewage, and farms, but can also be detected in other relatively pristine environments, including rivers, lakes and soils (52). Natural water bodies have been reported to act as significant environmental reservoirs for ARB and ARGs owing to the inherent density and diversity of bacterial loading (5, 6, 43).

The distribution and aggregation of AR is not just a case of inheritance or vertical gene transfer, as it occurs mainly due to horizontal gene transfer (HGT) (33, 39, 52). Horizontal transfer of ARGs is always facilitated by vehicles, including plasmids, transposons, integrons, and bacteriophages. Once ARGs are inserted into these mobile genetic platforms, they can be spread among various species and genera (10, 39, 50). There is clear evidence of the exchange of ARGs between environmental and clinical bacteria (52), and natural reservoirs of ARGs have long been considered as an unlimited source of transferable traits for emerging pathogenic organisms (6).

Beta-lactam antibiotic is one of the most broadly used antibiotic compounds (19). The most common mechanism of bacterial resistance to β-lactam antibiotics is the presence of extended-spectrum β-lactamases (ESBLs) along with plasmid-mediated AmpC β-lactamases, which are both capable of hydrolyzing these agents (8). Nearly all ESBLs originate from the common *TEM*, *SHV*, *OXA*, and *CTX-M* genes. In particular, these genes can also be horizontally transferred with mobile genetic elements. Moreover, ESBLs are even indicated to be strongly correlated with multidrug resistance in Enterobacteriaceae (40, 46).

China has long been considered to be the largest antibiotics producer and consumer in the world and it has been established that about 210,000 tons of antibiotics are produced annually, according to a 2007 survey (29). Additionally, 30% of drugs sold in Chinese hospitals and medical stores are antibiotics, while the proportion is only about 10% in the developed world (11). China also has the highest level of antibiotic resistance and, even worse, a higher rate of resistance development in comparative analysis with Kuwait and the United States (2). In China, many studies have reported the prevalence and characterization of AR in surface water or ground water and resistance appears to be spread rapidly in many regions (6, 18, 44).

Lake Taihu, a large shallow freshwater lake in China with an area of 2338 km^2^ (47), acts as a main source of drinking, irrigation and fishery water (53). With the extensive growth in agriculture and industry in the past few decades, Lake Taihu has been investigated for eutrophication with high loading of nitrogen and phosphorus, as well as a heavy density of water bloom (17, 35, 61). However, studies about antibiotic pollution and resistance in the lake are still scare. Recent studies have demonstrated the wide distribution of antibiotic resistance-associated genes, including tetracycline resistance genes (*tet*) and the class 1 integron gene (*Int* I) in the lake (60), and the presence of four ARG concentrations in lake sediments was in the following sequence: *strB* > *qnrB* > *strA* > *qnrS* (57), bringing up important issues for better understanding of the diversity and abundance of antibiotic resistance, and the potential of antibiotic resistance dissemination among the indigenous flora of this aquatic environment. Currently, culture-independent methods, such as metagenomic analysis, are widely applied to detect ARGs in natural water and wastewater treatment plants (WWTP) (20, 49). However, sometimes a discrepancy between genotype and phenotype may be caused by the bias in nucleic acid manipulation, and the isolation of antibiotic-resistant bacteria could help to illustrate pollution with ARB, ARGs expression and transfer potential directly.

In this paper, through a combination of culture-dependent approaches and polymerase chain reaction (PCR) methods, we aim to depict: 1) the antibiotic resistance profiles and the characteristics of AR in isolates recovered from nine disparate areas across Lake Taihu, as well as the correlation of various environment factors with antibiotic resistance; 2) the diversity and distribution of ESBL genes and integrase genes; and 3) the dissemination potential of transferable antibiotic resistance assessed through conjugation mating experiments.

## Materials and Methods

### Sampling and enumeration of total culturable bacteria and ARB

**Fig. 1:**
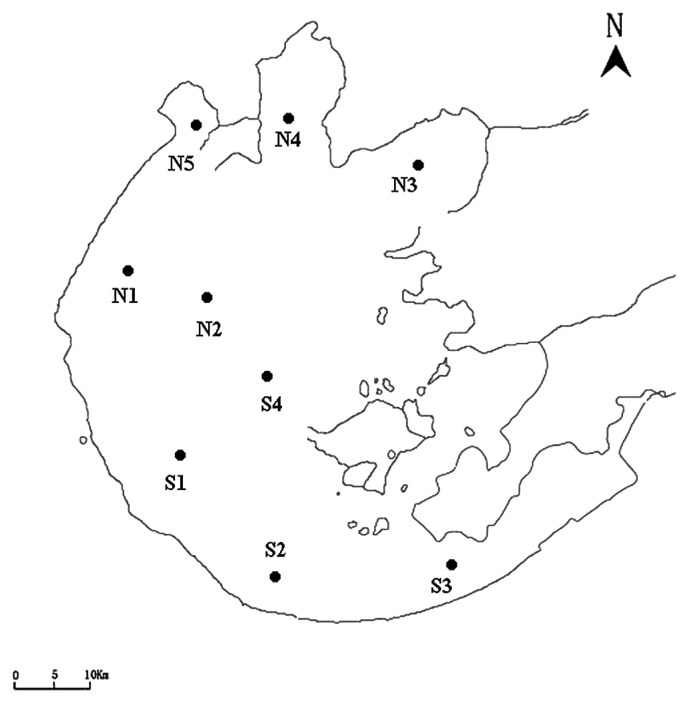
Location of sampling sites in Lake Taihu.

Nine sites across Lake Taihu were selected for water sampling on October 26, 2011 (Fig. 1): site S1 (31°06′56″ N, 120°00′33″ E), S2 (30°58′14″ N, 120°08′17″ E), S3 (30°58′53″ N, 120°16′21″ E), S4 (31°11′18″ N, 120°10′09″ E), N1 (31°18′14″ N, 119°58′17″ E), N2 (31°16′23″ N, 120°03′55″ E), N3 (31°24′00″ N, 120°20′13″ E), N4 (31°27′52″ N, 120°10′37″ E), N5 (3131°18′14″ N, 119°58′17″ E). The Luria-Bertani (LB) and LB supplemented with inhibitory concentrations of antibiotics were used to obtain the total culturable bacteria and ARB, respectively. All plates were incubated at 30°C for 24–48 h and viable cells were enumerated. The antibiotics used in this study were purchased from Sunshine (Nanjing, China), and were as follows: ampicillin (Amp), kanamycin (Km) and streptomycin (Str) were supplemented to a final concentration of 100 μg mL^−1^, while gentamicin (Gm), tetracycline (Tet) and chloramphenicol (Cm) were used at a final concentration of 20 μg mL^−1^.

### Bacteria isolation and identification of isolates

Isolates were grouped according to sampling sites and antimicrobial resistance patterns. Bacterial identifications were carried out using 16S rRNA gene sequence analysis. Briefly, a boiling method (1) was adopted for extraction of the complete DNA, and the primers 27F (5′-AGAGTTTGATCCTGGCTCAG-3′) and 1492R (5′-GGTTACCTTGTTACGACTT-3′) (Table 1) were used to amplify the bacterial 16S rRNA gene (16). Then the near-complete 16S rRNA gene was digested with *Hha* I (Takara Bio, Otsu, Japan) and grouped through restriction fragment length polymorphism (RFLP) analysis (54). At least one representative isolate from each group was sequenced by Beijing Genomics Institute (BGI). Online similarity searches were conducted with BLAST software at the National Center of Biotechnology Information (NCBI) website.

### Antimicrobial sensitivity testing

Antimicrobial susceptibility testing was carried out by the 1% proportion method according to the laboratory’s standard procedure (9). Briefly, isolated colonies of each test strain were first picked from the plates grown overnight, inoculated into 5 mL LB broth and incubated overnight at 30°C, and diluted to a final cell density of about 1×10^4^ CFU mL^−1^ in phosphate-buffered saline (PBS). Next, 100 μL sample of the suspension was spread on LB agar plates containing inhibitory concentrations of antibiotics just as described above in the *Sampling and enumeration of total culturable bacteria and ARB* section and controls. The inoculated plates were incubated at 30°C for 24–48 h and viable cells were enumerated. Antibiotic sensitivity/resistance patterns were determined according to the inhibition of macroscopic growth (*e.g.*, a sensitive isolate has <1% resistant population whereas a resistant isolate has >1%) (9). *Escherichia coli* (*E. coli*) MG1655 strain was used as the quality control strain in antimicrobial susceptibility testing.

### Identification of β-lactamase genes and integrons

Primers used to amplify the major members of the β-lactamase genes, other plasmid-mediated AmpC and specific integrase genes are listed in Table 1. For each isolate, the PCR reaction mixtures (20 μL) contained 0.2 mM of each dNTP, 0.1 μM of forward and reverse primers (BGI, China), 1 U Ex *Taq* DNA polymerase (Takara Bio), and 40 ng bacterial DNA. All PCR products were visualized by electrophoresis on 1.0% (w/v) agarose gels stained with ethidium bromide.

### Conjugation mating experiments

Conjugation was carried out by the membrane filter mating assay (34) using *E. coli* Top10 strain (Str^r^) and *E. coli* SM10 strain (Km^r^) as the recipient strains. Briefly, the donor and recipient bacteria were inoculated in LB broth and grown to the logarithmic phase, mixed at a 1:1 ratio (v/v), spotted on a 0.22 μm filtration membrane and incubated at 37°C for 10 h. Conjugants were selected on LB agar plates supplemented with a combination of 100 μg mL^−1^ kanamycin or 100 μg mL^−1^ streptomycin and one of the following other antibiotic compounds: ampicillin (Amp) (100 μg mL^−1^), gentamicin (Gm) (20 μg mL^−1^), tetracycline (Tet) (20 μg mL^−1^), or chloramphenicol (Cm) (20 μg mL^−1^). Antibiotic sensitivity/resistance patterns of conjugants were determined as described above in the *Antimicrobial sensitivity testing* section.

### Data analysis

Canonical correspondence analysis (CCA) was carried out using Canoco for Windows software (version 4.5) and was used to explore the influence of selected environmental variables on antibiotic resistance rates at different sites. All parameters were log_10_-tranformed to ensure normal distribution and standardized. The significance of the relationship between ARB populations and environmental variables was assessed using Monte Carlo permutation tests.

### Nucleotide sequence accession numbers

The 16S rRNA gene nucleotides sequences reported in the current study have been deposited in the GenBank database under accession numbers KC139681–KC139702, and KC161201–KC161203.

## Results

### Antibiotic resistance profiles of Lake Taihu

**Fig. 2:**
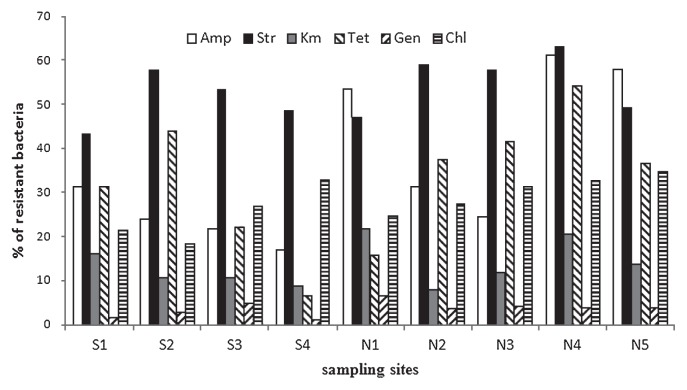
Distribution of antibiotic susceptibility in the isolated strains recovered from nine sampling sites across Lake Taihu.

Overall, the number of isolates recovered on LB plates from the 9 sites was 3.2×10^3^ CFU mL^−1^ on average and no statistically significant differences were observed among them. These culturable bacteria showed low frequency of resistance to kanamycin and gentamicin, while high levels of resistance to ampicillin (17.0%–61.3%) and streptomycin (43.2–63.1%) were detected, followed by tetracycline and chloramphenicol (Fig. 2). The frequencies of resistance to the three aminoglycosides antibiotics, streptomycin, gentamicin and kanamycin, were relatively constant in all 9 sites, ranging from 43.2% to 63.1%, 1.0% to 6.0% and 6.0% to 21.0%, respectively. By contrast, resistance to ampicillin exhibited obvious spatial heterogeneity. In the present study, over 50.0% of the isolates obtained in N1, N4 and N5 were resistant to ampicillin, whereas only 20.0%–30.0% of isolates from other sites exhibited ampicillin resistance.

**Fig. 3:**
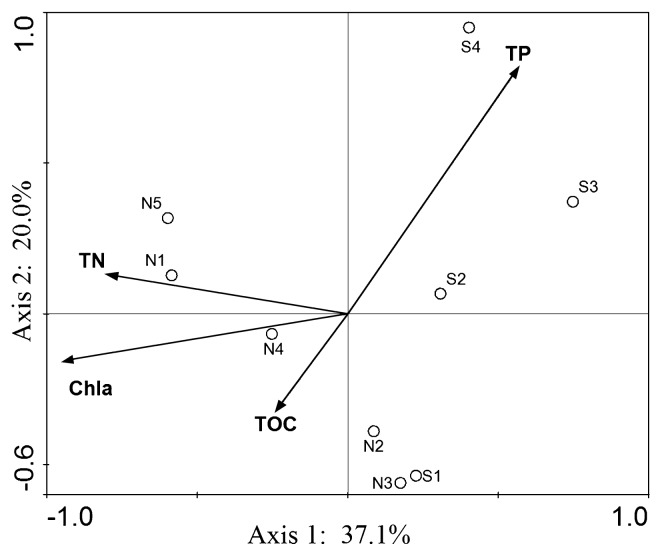
Canonical correspondence analysis (CCA) compares the abundance of tested resistance bacteria (symbols) and the environmental factors. TP, total phosphorus; TN, total nitrogen; TOC, total organic carbon; Chl *a*, chlorophyll *a*. Circles represent different sampling sites.

CCA analysis was used to correlate the effect of selected water chemical properties on antibiotic resistance patterns, and the results revealed a significant correlation between the AR variation and environmental factors. A total of 57.1% variations could be explained by the selected environmental factors (Fig. 3). N1, N4 and N5 clustered together and were strongly affected by Chl *a* and TN.

### Diversity and antimicrobial susceptibility of the isolated antibiotic-resistant bacteria

Seventy-eight bacterial isolates were randomly selected for further study, were classified into 11 groups based on RFLP analysis of the 16S rRNA gene, and 24 representative isolates were selected for sequencing (Table 2). According to the DNA sequences, representative isolates from each group were affiliated to the same genus, indicating the reliability of RFLP classifications. It was found that the genera of *Pseudomonas* (35.9%) and *Acinetobacter* (20.5%) dominated in the 78 isolates, followed by *Agrobacterium* (9.0%), *Stenotrophomonas* (7.7%), *Bacillus* (5.1%), *Brevundimonas* (5.1%), *Microbacterium* (5.1%), *Comamonas* (5.1%), *Cupriavidus* (2.6%), *Flavobacterium* (2.6%), and *Sphingomonas* (1.3%). Ninety percent of the isolates were Gram-negative and most of the isolates were indigenous microorganisms found in freshwater environments. In particular, among the 28 *Pseudomonas* strains, 48.5% were affiliated with *P. aeruginosa* through colony characteristics, which is known as a potential pathogen.

In agreement with the resistance profiles across the lake, high frequencies of resistance to ampicillin and streptomycin were also observed among these strains selected for further study. Multiple antibiotic resistance (MAR), that is, exhibiting resistance to three or more antibiotics, was observed in 62.0% of the isolates. The occurrence of an antibiotic phenotype was mainly related with the taxonomic affiliation of the organisms (Table S1). The genera *Brevundimonas* and *Comamonas* showed sensitivity to gentamicin. The most frequent resistance profile among *P. aeruginosa* isolates was AMP-KM-TET-CHL, indicating the cross resistance of the four antibiotics. Among *Acinetobacter* isolates, the most frequent resistance pattern was AMP-STR-KM-GEN. In particular, *Acinetobacter* isolates showed a higher level of resistance to gentamicin.

### Diversity of β-lactamase genes and integron genes

The β-lactamase genes in the Amp-resistant strains, *i.e. bla_SHV_*, *bla_TEM_*, *bla_OXA-1_*, *bla_CTXM_*, and 6 other plasmid-mediated AmpC genes were screened by PCR using specific primers (Table 1). Thirty-one of the 64 Amp-resistant isolates were found to carry at least one of these β-lactamase genes. The most predominant genotype detected was *bla_TEM_* (22.0%), followed by *bla_SHV_* (12.5%), *bla_CTXM_* (7.8%) and *bla_OXA-1_* (1.6%). As for the plasmid-mediated AmpC β-lactamase genes, only *bla_EBCM_* and *bla_MOXM_* were detected in 4 and 3 isolates, respectively. The distributions of β-lactamase genes were different in each species/genus. Of the 28 *Pseudomonas* isolates, only *bla_TEM_* gene and *bla_SHV_* were detected in 6 and 2 isolates, respectively, although Amp resistance presented as the main phenotype. In the *Acinetobacter* isolates, 2 *bla_TEM_*, 2 *bla_SHV_*, 1 *bla_OXA-1_* and 3 *bla_CTXM_* were detected. As for the other minority genera, *Agrobacterium* and *Stenotrophomonas* also demonstrated the prevalence of *bla_TEM_* and *bla_SHV_*.

Thirty (38.5%) of the 78 isolates were found to carry *int* I, of which 3 isolates harbored both *int* I and *int* II. *Int* III, representing the class 3 integrons, was not detected in the present study (Table 3). Among all isolates tested, relatively high proportions of integrons were detected in *Pseudomonas* (16/28, 57.1%), *Stenotrophomonas* (3/6, 50.0%), *Bacillus* (2/4, 50.0%), *Cupriavidus* (1/2, 50.0%) and *Comamonas* (2/4, 50.0%), followed by 31.3% of *Acinetobacter* (5/16) and lastly 14.3% (1/7) of *Agrobacterium*. No integrons were detected in *Brevundimonas*, *Microbacterium*, *Flavobacterium*, and *Sphingomonas*. In particular, in *P. aeruginosa* isolates, up to 83.3% (10/12) were observed to carry the *int* I gene. About 28 (93.3%) of the 30 integron-positive strains were detected to show multiple antibiotic resistance, while the proportion in all the integron-negative strains was only 53.7%. Thus, the presence of integron may enable better prediction of antibiotic resistance.

### Conjugation of antibiotic resistance

For the use of streptomycin or kanamycin as selective markers, only 63 isolates with only one of the two antibiotic resistances could be subjected to conjugation assays as donor cells. Overall, 40 strains, distributed in 9 genera, were successfully able to transfer antibiotic resistance to *E. coli* SM10 or *E.coli* Top10 through conjugation (Table 4). Among them, only 9 conjugants exhibited all resistance profiles of the donor strains. In terms of the conjugation frequencies of different antimicrobial resistance, the spread of ampicillin (58.3%) and tetracycline resistance (57.1%) was quite high, reaching almost 3 times that of streptomycin (20.1%) and gentamicin (15.4%). As expected, the conjugation frequency also showed a positive correlation with the presence of integrase genes, and among the 40 strains that successfully transferred MAR to recipient cells, 22 possessed at least one type of integrase gene and 26 phenotypically demonstrated MAR.

## Discussion

Our study demonstrated the significant prevalence of antibiotic-resistant bacteria in the surface water of Lake Taihu. To our knowledge, these are the first data to document thoroughly the breadth and spread of antibiotic resistance against some commonly used antibiotics in this lake region. Although the prevalence of ARB in many freshwater sources has been well documented (21, 42), it was surprising for the high frequency of ampicillin and streptomycin resistance found in Lake Taihu, especially in the western and northern sites of N1, N4 and N5. A recent study of the antibiotics in the surface water of Yangtze Estuary showed that the dissolved concentrations of all target antibiotics were in the ng L^−1^ level (59), much lower than the minimum inhibition concentration (MIC) of the ARB. Meanwhile, the concentrations detected for β-lactams always remained low in spite of its extensive use, due to low stability and persistence (12, 13, 22). As one of the most densely populated areas with large amounts of antibiotics used in human medicine, animal farming, and agriculture in this region, previous analysis of water and sediment from Lake Taihu illustrated the prevalence of tetracycline-resistant genes (60). A recent study also reported that *E. coli* from Lake Taihu sediment possessed higher resistance to streptomycin, tetracycline and ampicillin than four other antibiotics (57), which was consistent with our results. It has been indicated that the antibiotic compounds found in water were responsible for the emergence and dissemination of antibiotic-resistant bacteria, even when they were much lower than the minimal inhibitory concentration (MIC) (26, 37). However, it is not objective to explain the antibiotic resistance phenotype only based on antibiotic concentration. In the present study, the sites with higher resistance frequency, N1, N4 and N5, are located in a heavily polluted lake area with influent from nearby cities, and antibiotics or ARB carried by discharged sewage may contribute to antibiotic resistance. It has been reported that the chlorination process in sewage treatment can also contribute to the selection of ARB (56), and some β-lactam-resistant genes (*bla_TEM_* and AmpC genes) could be enriched through chlorination (49). In addition, our results demonstrated that Amp resistance displayed the highest transfer potential among all the detected antibiotics (Table 4), and Amp resistance in site N1, N4 and N5 may be accelerated owing to the high level of nutrients (28). As can be seen from CCA analysis, the antibiotic resistance profile of N1, N4 and N5 clustered together and was separate from other sites (Fig. 3), mainly on the first axis, which correlated with the Chl *a* and TN concentration, indicating that Chl *a* and TN could be important factors affecting the levels and distributions of antibiotic resistance in this aquatic environment. However, further study on both phenotypic and molecular scales are required to identify to what extent antibiotic resistance is linked to these anthropogenic-driven selective pressures.

Seventy-eight strains, dominated by *Gammaproteobacteria* of *Pseudomonas* and *Acinetobacter*, were randomly selected for further analysis. In our other study of culture-independent analysis of the bacterioplankton community through 16S rRNA gene clone library and T-RFLP, *Betaproteobacteria*, *Actinobacteria* and *Alphaproteobacteria* were the three major groups (unpublished data), indicating the significant superiority of *Pseudomonas* and *Acinetobacter* in the ARB population. *Pseuodomonas*, colonizers of the aquatic environment, possess pronounced capacity for the acquisition and dissemination of resistance genes, and strains belonging to this genus are in fact frequently resistant to several antimicrobial agents, with susceptibility patterns similar to those of clinical strains (38). It is noticeable that *P. areuginosa* and *Acinetobacter* are both important opportunistic pathogens responsible for a variety of nosocomial infections, and other minority isolates are also phylogenetically related to opportunistic or nosocomial pathogens, including *Cupriavidus respiraculi* (14), *Sphingomonas* (45) and *Flavobacterium* (23). Considering that 62.0% of the 78 isolates were identified as multiple ARBs, the presence of opportunistic pathogens that exhibit resistance to diverse antibiotics may be serious hazards for consumers of lake water (48).

Amp resistance was overwhelming in culturable ARB across Lake Taihu and also among the 78 isolates. Beta-lactamases are ancient enzymes originally encoded in bacterial chromosomes (8). Recently, ESBL-producing bacteria have been rapidly spreading throughout the world (8) and constitute a serious threat to human health (40, 46). Our results showed that the ESBL genes *bla_TEM_* and *bla_SHV_* had significantly higher frequency among the 78 isolates, followed by *bla_CTXM_* and *bla_OXA-1_*, indicating the prevalence of ESBLs in Lake Taihu isolates. Plasmid-mediated *bla_TEM_*, mostly found in *E. coli* and *K. pneumoniae* (31, 32), has been reported to dominate the lactam-resistant genes and is frequently detected in natural aquatic systems (4, 27). Our results also revealed that *bla_TEM_* can be detected in Gram-positive *Bacillus*, probably due to gene transfer in the aquatic environment. New families of ESBLs, such as *CTXM* and *OXA-1* types, were found in our study. For example, *CTXMs* were also found in our environmental isolates, including *Acinetobacter*, *Agrobacterium* and *Brevundimonas*. In recent years, *bla_CTXM_* has gradually become the most widespread class (15) and it has mainly been found in strains of *Salmonella enterica* serovar Typhimurium, *E. coli* and other species of *Enterobacteriaceae* (8). The detected AmpC genes in our environmental isolate have been reported to be mobilized to a substantial degree on plasmids only in the last few decades in clinical settings (30). In general, it can be concluded that new families of human-associated ESBLs and AmpC genes can also be found in natural environmental isolates.

Consistent with a previous study (55), the observation of antibiotic- and beta-lactamase class-specific resistance genes distribution in the culturable bacteria in this study also indicates that integron-positive isolates are more likely to be antibiotic resistant and even multidrug resistant. In particular, the predominantly class 1 integrons were found in all integron-positive ARB. Tacão *et al.* (51) showed that >50% of the environmental bacterial isolates contained class 1 integron. Zhang *et al.* (60) also reported that each water sample contained a significant number of the class 1 integron (10^3^ copies mL^−1^) in Lake Taihu. Integrons are known to play a major role in the introduction and spread of antibiotic resistance genes in environmental bacteria due to their ability to capture and exchange genes via site-specific recombination (55, 25, 36). For further investigation, conjugation, a gene horizontal transfer path, was examined. In the conjugation study, 63.5% of the bacteria were able to transfer an antibiotic-resistant gene to *E. coli*, highlighting the high frequency of antibiotic resistance-associated gene dissemination in Lake Taihu. These results provide evidence that a wide variety of clinically important antibiotic resistance genes are mobile within aquatic bacterial communities. However, a few conjugants shared the same antibiotic resistance profiles with the donor strains, while the others just acquired part of the antibiotic resistances of donors. This can be interpreted that only ARGs that are situated in mobile genetic platforms, such as plasmids, transposons, integrons, or bacteriophages, can be horizontally transferred across cells (36). All of the examined *Int* I-positive isolates could transfer antibiotic resistance. Given the high level of *Int* I detected in Lake Taihu in a previous study (60), it is assumed that uncultured bacteria also constitute reservoirs for antibiotic resistance genes in natural systems. Additionally, aquatic organisms from phytoplankton to large aquatic mammals could act as vectors to further facilitate the transmission of microorganisms and meanwhile represent an important environmental matrix within which HGT can take place (7, 24). Combined together, the frequency of HGT in freshwater, for example Lake Taihu, may be higher than expected. Moreover, considering that *E. coli* strains acted as recipients in the assay, our results confirmed the flow of resistance genes between native and foreign organisms and indicated the possibility of ARG transfer from environmental reservoirs to clinical pathogenic strains, which should be underlined in the future.

In summary, we have reported a comprehensive study of antibiotic resistance in Lake Taihu and the results revealed that a pool of antibiotic-resistant bacteria and associated genes existed in the surface water. Resistance against ampicillin and streptomycin occurred in high frequency, especially in western and northern regions of Lake Taihu. CCA analysis revealed that some environmental factors, including Chl *a* and TN, might be important to exert positive influences on the variations in antibiotic-resistant populations. New families of human-associated ESBLs and AmpC genes can be found in natural environmental isolates. The distribution of integrons and the horizontal transfer of ARGs across different genera indicated the prevalence of the promiscuous exchange and communication of genes within the large, shallow lake. The prevalence of AR and the dissemination of transferable antibiotic resistance in bacterial isolates (especially pathogenic bacteria) call for further studies to determine the extent to which the dissemination of antibiotic-resistant bacteria occurs and the health risks that this dissemination poses by invading human and animal populations who are dependent on the lake for water consumption.

## Supplementary Information



## Figures and Tables

**Table 1:** PCR primers used in this study

Primer	Target	Sequence (5′–3′)	Amplicon size (bp)	Reference
27F	16S rRNA gene	AGAGTTTGATCCTGGCTCAG	1465	
1492R		GGTTACCTTGTTACGACTT		16
SHV-U	*bla_SHV_*	AGGATTGACTGCCTTTTTGCGCC	345	
SHV-D		ATCACCACAATGCGCTCTGCT		55
TEM-U	*bla_TEM_*	AGTTCTGCTATGTGGTGCGG	481	
TEM-D		ATCAGCAATAAACCAGCCAGCC		55
OXA-1-U	*bla_OXA-1_*	GTGCGTCAACGGATATCTCT	736	
OXA-1-D		GTGATCGCATTTTTCTTGGC		55
CTXM-U	*bla_CTXM_*	TCCCAGAATAAGGAATCCCAT	479	
CTXM-D		CCCATTCCGTTTCCGCTA		15
MOXM-U	*MOX-1, MOX-2, CMY-2*	GCTGCTCAAGGAGCACAGGAT	520	
MOXM-D	*CMY-8 to CMY-11*	CACATTGACATAGGTGTGGTGC		55
CITM-U	*LAT-1 to LAT-4, CMY-2*	TGGCCAGAACTGACAGGCAAA	462	
CITM-D	*CMY-7, BIL-1*	TTTCTCCTGAACGTGGCTGGC		55
DHAM-U	*DHA-1, DHA-2*	AACTTTCACAGGTGTGCTGGGT	405	
DHAM-D		CCGTACGCATACTGGCTTTGC		55
ACCM-U	*ACC*	AACAGCCTCAGCAGCCGGTTA	346	
ACCM-D		TTCGCCGCAATCATCCCTAGC		55
EBCM-U	*MIR-1, ACT-1*	TCGGTAAAGCCGATGTTGCGG	320	
EBCM-D		CTTCCACTGCGGCTGCCAGTT		55
FOXM-U	*FOX-1 to FOX-5*	AACATGGGGTATCAGGGAGATG	190	
FOXM-D		CAAAGCGCGTAACCGGATTGG		55
Int I-U	Class 1 integrase gene	ACGAGCGCAAGGTTTCGGT	565	
Int I-D		GAAAGGTCTGGTCATACATG		This study
Int II-U	Class 2 integrase gene	GTGCAACGCATTTTGCAGG	403	
Int II-D		CAACGGAGTCATGCAGATG		This study
Int III-U	Class 3 integrase gene	CATTTGTGTTGTGGACGGC	717	
Int III-D		GACAGATACGTGTTTGGCAA		This study

**Table 2:** Distribution of genera among the 78 strains isolated from Lake Taihu and the 24 representative isolates selected for sequencing

Genus	No. of isolates (%)	No. of representative isolates
*Pseudomonas* spp.	28 (35.9)	7
*Acinetobacter* spp.	16 (20.5)	5
*Agrobacterium* spp.	7 (9.0)	2
*Stenotrophomonas* spp.	6 (7.7)	1
*Bacillus* spp.	4 (5.1)	1
*Brevundimonas* spp.	4 (5.1)	2
*Microbacterium* spp.	4 (5.1)	2
*Comamonas* spp.	4 (5.1)	1
*Cupriavidus* spp.	2 (2.6)	1
*Flavobacterium* spp.	2 (2.6)	1
*Sphingomonas* spp.	1 (1.3)	1

**Table 3:** Distribution of various ARB genes among different species/genera

Species/Genus	No. of Bla Genotypes (% a)						No. of Integrases (%^b^)		
	
	TEM	SHV	OXA-1-1	CTXM	ECBM	MOXM	Int I	Int II	Int III
*P. aeruginosa*	1 (1.6)	2 (3.1)	0	0	0	0	10 (12.8)	1 (1.3)	0
*Pseudomonas*	6 (9.5)	2 (3.1)	0	0	0	0	16 (20.5)	2 (2.6)	0
*Acinetobacter*	2 (3.1)	2 (3.1)	1 (1.6)	3 (4.7)	0	0	5 (6.4)	0	0
*Agrobacterium*	2 (3.1)	1 (1.6)	0	1 (1.6)	0	0	1 (1.3)	0	0
*Stenotrophomonas*	2 (3.1)	1 (1.6)	0	0	1 (16.7)	1 (1.6)	3 (3.8)	0	0
*Bacillus*	1 (1.6)	0	0	0	1 (25.0)	1 (1.6)	2 (2.6)	0	0
*Brevundimonas*	0	0	0	1 (1.6)	0	0	0	0	0
*Microbacterium*	0	0	0	0	0	1 (1.6)	0	0	0
*Comamonas*	0	0	0	0	2 (50.0)	0	2 (2.6)	1 (1.3)	0
*Cupriavidus*	0	1 (1.6)	0	0	0	0	1 (1.3)	0	0
*Flavobacterium*	0	0	0	0	0	0	0	0	0
*Sphingomonas*	1 (1.6)	0	0	0	0	0	0	0	0
Total	14 (22.0)	7 (12.5)	1 (1.6)	5 (7.9)	4 (6.3)	3 (4.8)	30 (38.5)	3 (3.9)	0

a Percentage of strains containing β-lactamase genes in the 64 Amp-resistant isolates.

b Percentage of integron-positive strains among all 78 screening isolates.

**Table 4:** Antimicrobial resistance patterns of donor strains and conjugants

Genus	Isolates*	Donor resistance profile	Resistance patterns of the conjugants
*Pseudomonas*	S1-A3	AMP-CHL	CHL
	S1-K3	STR-KM	KM
	S1-A5	AMP-KM-CHL	AMP-KM-CHL
	S1-T4	AMP-KM-TET-CHL	AMP
	S2-A6	AMP-TET-CHL	AMP
	S2-T8	AMP-STR-TET-CHL	AMP-STR
	S3-P1	AMP-STR-TET-CHL	AMP-TET-CHL
	S4-A15	AMP-STR-CHL	AMP-STR-CHL
	S4-T13	AMP-STR-KM-TET-CHL	AMP, STR
	N1-A24	AMP-STR-KM	AMP-KM
	N2-A27	AMP-KM-TET-CHL	AMP-KM-TET-CHL
	N2-A29	AMP-KM-TET-CHL	AMP-KM-TET-TET
	N2-A30	AMP-TET-CHL	AMP-TET
	N4-A32	AMP-STR-TET-CHL	AMP-CHL
	N5-P7	AMP-TET-CHL	AMP-TET
*Acinetobacter*	S1-A2	AMP-STR-CHL	STR-CHL
	S1-K2	STR-KM	KM
	S2-A10	AMP-STR-KM-GEN	AMP
	S3-A11	AMP-STR	AMP
	S3-A12	AMP-STR-GEN-CHL	AMP-CHL
	S4-A17	AMP-GEN-CHL	GEN-CHL
	N4-A33	AMP-TET-CHL	AMP-TET-CHL
	N4-A37	AMP-STR-TET-GEN-CHL	STR-TET
*Agrobacterium*	S1-A4	AMP-KM-CHL	CHL
	S1-K1	KM-CHL	KM-CHL
	N4-A35	AMP-KM-TET-GEN-CHL	AMP-KM-TET-CHL
	N4-A36	AMP-STR-CHL	AMP
*Comamonas*	S2-A9	AMP-STR	AMP
	S3-A13	AMP	AMP
	S3-A14	AMP-STR-CHL	CHL
*Brevundimonas*	S2-A7	AMP	AMP
	S4-A19	AMP-STR-GEN-CHL	STR-GEN
*Stenotrophomonas*	S3-T12	AMP-TET	AMP
	N5-P1	AMP-TET-CHL	AMP-TET-CHL
*Cupriavidus*	N1-A25	AMP-STR-KM-GEN	STR-KM-GEN
	N4-A38	AMP-CHL	AMP
*Microbacterium*	N2-A28	AMP-CHL	AMP-CHL
	N2-K10	KM	KM
*Bacillus*	N5-P3	AMP-CHL	AMP-CHL
	N5-P6	AMP-KM-TET-GEN-CHL	AMP

* Isolates were named according to sampling sites.
