# Supplementary material for: Occurrence and Distribution of Antibiotic-resistant Bacteria and Transfer of Resistance Genes in Lake Taihu
Source: Microbes Environ. 2013 Nov 16;28(4):479–86. doi: 10.1264/jsme2.ME13098 (PMC4070710; doi:10.1264/jsme2.ME13098)
Supplement: Supplementary file 1 [file 28_479_s1.pdf]

Supplementary Material

Occurrence and distribution of antibiotic-resistant bacteria and transfer of resistance genes in Lake Taihu

Qian Yin, Dongmei Yue, Yuke Peng, Ying Liu, Lin Xiao*

Number of pages : 1

Number of tables: 1

Table S1 – AMR patterns among *Pseudomonas* and *Acinetobacter*

Species/Genus	AMR pattern	n(%)
	AMP-KM-TET-CHL	6(7.7)
P. aeruginosa	AMP-KM-TET-CHL-STR	3(3.8)
	AMP-STR-TET-CHL	2(2.6)
	AMP-CHL	1(1.3)
P. Nitroreducens	AMP-KM-CHL	1(1.3)
	AMP-STR-CHL	1(1.3)
	AMP-TET-CHL	4(5.2)
	AMP-TET	1(1.3)
P. mendocina	AMP	1(1.3)
	AMP-STR-KM	1(1.3)
	AMP-STR	2(2.6)
	AMP-STR-TET-CHL	1(1.3)
P. Stutzeri	KM-CHL	1(1.3)
	AMP-KM-GEN-CHL	1(1.3)
P. pseudoalcaligenes	AMP-TET	2(2.6)
	AMP-STR-KM-GEN	3(3.8)
	AMP-TET-GEN	2(2.6)
	CHL	1(1.3)
	AMP-TET-CHL	1(1.3)
***Acinetobacter* spp.**	AMP-STR-TET-CHL	1(1.3)
	AMP-STR-GEN-CHL	1(1.3)
	AMP-CHL-GEN	2(2.6)
	AMP-GEN	1(1.3)
